# What High-Income States Should Do to Address Industrial Antibiotic Pollution

**DOI:** 10.1093/phe/phaa020

**Published:** 2020-09-02

**Authors:** Erik Malmqvist, Christian Munthe

**Affiliations:** University of Gothenburg

## Abstract

Antibiotic resistance is widely recognized as a major threat to public health and healthcare systems worldwide. Recent research suggests that pollution from antibiotics manufacturing is an important driver of resistance development. Using Sweden as an example, this article considers how industrial antibiotic pollution might be addressed by public actors who are in a position to influence the distribution and use of antibiotics in high-income countries with publicly funded health systems. We identify a number of opportunities for these actors to incentivize industry to increase sustainability in antibiotics production. However, we also show that each alternative would create tensions with other significant policy goals, necessitating trade-offs. Since justifiable trade-offs require ethical consideration, we identify and explore the main underlying normative issues, namely, the weighing of local versus global health interests, the weighing of present versus future health interests, and the role of individualistic constraints on the pursuit of collective goals. Based on this analysis, we conclude that the actors have weighty principled reasons for prioritizing the goal of addressing pollution, but that translating this stance into concrete policy requires accommodating significant pragmatic challenges.

## Introduction

Antibiotic resistance (ABR) is widely recognized as a major threat to public health and healthcare systems worldwide. The diminishing effectiveness of antibiotics undermines our ability to treat bacterial infections and jeopardizes important areas of modern medicine such as surgery, organ transplantation and cancer care (Laxminarayan *et al.*, 2013). The economic and developmental impact of ABR is profound and is expected to grow significantly, especially in the developing world (World Bank, 2017; ReAct, 2019). Many factors contribute to ABR, including insufficient prevention and control of infections, appropriate as well as inappropriate antibiotic consumption, and liberal antibiotic use in animal farming. In addition, recent research highlights the role played by industrial emissions of antibiotics into the environment, primarily through wastewater from production of active pharmaceutical ingredients (APIs) in countries such as India and China (Larsson, 2014; Larsson *et al.*, 2018).^1^

The need to address industrial pollution to curb ABR is recognized by scholars, non-governmental organizations, governments and the pharmaceutical industry itself, and different strategies for accomplishing this goal have been proposed (Laxminarayan *et al.*, 2013; Ågerstrand *et al.*, 2015; AMF, 2017; AMR Industry Alliance, 2018; Bengtsson-Palme *et al.*, 2018; Nijsingh *et al.*, 2019;  WHO/FAO/OIE, 2020). However, all management approaches face significant challenges. For instance, legal hurdles, financial costs and reputational risks constitute powerful disincentives for pharmaceutical companies to increase supply chain transparency and exert control over subcontracted API producers (Nijsingh *et al.*, 2019). Moreover, API discharges are unregulated in most countries (Larsson, 2014) and governments in major API producer countries often have limited ability to enforce regulations due to e.g. corruption (Bengtsson-Palme *et al.*, 2018). More generally, the chain from API production to final antibiotics consumption involves many different public and private actors with widely diverging interests, creating uncertainty about how positive change could be incentivized. Nevertheless, a recent analysis has argued that responsibility and opportunities for action can be found in high-income states, where institutional systems surrounding pharmaceutical markets may be used to incentivize industry to move toward cleaner antibiotics production (Nijsingh *et al.*, 2019). In this article, we explore these opportunities from an ethical perspective in order to analyze what is required to design justifiable and feasible action.

We proceed as follows. First, we argue that public actors in high-income states indeed have weighty moral reasons to use their influence over industry to have them tackle production-related antibiotic pollution, before outlining how this may be accomplished by introducing environmental criteria into specific institutional functions of healthcare systems. We then demonstrate how each such approach conflicts with some other significant health policy goal, necessitating potentially controversial trade-offs. Making such trade-offs requires considering underlying normative conflicts as well as feasibility constraints, which we then identify and explore. We conclude by indicating areas requiring further inquiry.

Throughout the article, we focus on public actors in high-income countries with publicly funded health systems. We use Sweden as an example, but as other such countries have similar institutional functions (albeit the relevant institutional systems may be organized differently) our findings will apply more broadly. We will, moreover, speak of ‘environmental criteria’ in an intentionally broad sense. First, these criteria may have different *content*, including documented abidance by environmental laws in production countries, disclosure of the identity or geographic location of subcontracted producers, disclosure of producers’ emission levels and documented compliance with emission limits (Ågerstrand *et al.*, 2015; AMF, 2017; Bengtsson-Palme *et al.*, 2018). Which of these that are actually feasible, efficient and otherwise appropriate to use for incentivizing commercial actors is an unsettled issue; hence, our discussion does not presuppose any specific alternative. Second, the environmental criteria may perform different *roles*. They could work as *conditions of eligibility*, setting a threshold that suppliers must pass in order to be at all considered for a certain institutional action (authorization, procurement or subsidy). Alternatively, the criteria could work as *gradual weights*, to be balanced against other considerations of importance for the institutional action in question. Suppliers who fulfill environmental criteria are then (to varying degrees) favored in the competition with suppliers who do not (and are correspondingly disfavored), but neither is excluded from consideration. This distinction is important to our analysis and we return to it throughout the article.

## The Case for Addressing Pollution

At least three reasons can be given for why high-income state actors should take action to address industrial antibiotic pollution in producer countries, each reflecting a distinct widely recognized principle for allocating responsibilities to remediate large-scale collective harms (Miller, 2001). While these reasons are different, they may nevertheless complement each other to produce a stronger joint case than each can do individually. Moreover, should any of the reasons be found implausible, the others still provide substantial ground for action.

The first reason, reflecting the *capacity* principle (Singer, 1972; Miller, 2001), has already been hinted at: the actors in question are uniquely positioned to influence industry to reduce pollution. Given the magnitude of the problem and the difficulties facing other approaches, this capacity alone provides some reason for action.

The second reason reflects the *community* principle (Miller, 2001). Simplifying somewhat, one overarching goal of the actors is to protect and promote the health of a designated population. ABR jeopardizes the pursuit of this goal because it threatens to severely undermine the functioning of health systems. At the same time, the problem is global: resistance of bacteria in one location is capable of spreading quickly around the world. Thus, based on their concern for local public health, high-income state actors have reason to contribute to curbing resistance development also beyond their own countries’ borders.

The third reason appeals to the *moral responsibility* principle (Miller, 2001; Pogge, 2008). In economic parlance, antibiotic pollution represents a *negative externality*, i.e. a cost that is not borne by the parties to a transaction but that is instead shifted to a third party. The ‘cost’ here is significant: an increased risk of serious infections that cannot be effectively treated. Moreover, it is imposed on populations in producer countries and ultimately the global community at large without their consent. The pollution is directly caused by API producers, but these respond to a demand from buyers, in particular, health systems in high-income countries. While the precise role of specific actors within these systems in causing the pollution is difficult to conclusively determine, the priorities they make, particularly their effort to control costs, clearly generate a systematic demand for unsustainably produced antibiotics (Bengtsson-Palme *et al.*, 2018; Nijsingh *et al.*, 2019). In this way, these actors actively abet the serious harming of others without their consent, generating a strong reason to reduce their involvement in causing the harm and instead act to mitigate it.^2^

## Opportunities for Action

Several institutional functions are involved in determining how pharmaceuticals are made available, distributed and used within publicly funded healthcare systems in high-income countries. Environmental criteria can in principle be introduced into any of these functions in order to influence suppliers of antibiotics (be they research-based pharmaceutical firms, generic companies or parallel importers) to address production-related pollution. Using Sweden as an example, this section provides an overview of the relevant functions and considers how such criteria might work in them, highlighting the difference between using the criteria as eligibility conditions and as gradual weights (see above).^3^

In Sweden, the relevant institutional functions, their roles and the relationships between them can be briefly described as follows.

- *Authorization*: Selling a drug or medical product on the Swedish market requires authorization from an appropriate medical products agency (the Swedish *Läkemedelsverket,* LV, or the European Union’s *European Medicines Agency*, EMA).
- *Procurement*: Drugs used in inpatient care (representing 20 per cent of the total Swedish drug market in 2019 [TLV, 2020a]) are procured by the county councils that run the Swedish national healthcare service, using jointly agreed criteria for priority setting, fair competition and economic effectiveness, and with possible support from the National Agency for Public Procurement (*Upphandlingsmyndigheten*, UM).
- *Public Subsidy*: Drugs procured for inpatient care, and most prescription drugs distributed through pharmacies (including all antibiotics used in outpatient settings)—63 per cent of the total market—are subsidized by the government. Only 7 per cent of all prescription drugs sold lack any subsidy and are fully paid for by individual consumers. The Dental and Pharmaceutical Benefits Agency (*Tandvårds-och läkemedelsförmånsverket*, TLV) decides whether a drug receives subsidy, partly based on the supplier’s proposed price.
- *Generic Substitution*: The distribution of prescription drugs is governed by a generic substitution scheme, which legally requires pharmacies to offer consumers with prescriptions for off-patent branded drugs a selected cheaper generic substitute (SFS 2002:160, §21). Consumers can still get the branded drug but only by paying the difference in price between that drug and the substitute. LV assesses whether a generic is interchangeable with its branded counterpart; whereas TLV’s subsidy decisions determine which drug, among several interchangeable alternatives, consumers will be offered. In the Swedish setting, this function is thus closely connected to the subsidy function.

We will now consider each function in greater detail.

### Authorization

Since all new drugs must pass an authorization procedure to be licensed for marketing, licensing agencies have significant power to influence industry behavior, e.g. to incentivize companies to increase supply chain sustainability. In contexts where this mechanism is harmonized across national jurisdictions, e.g. the EU, this power does not lie with the national agency, but with the institution in charge of the harmonized authorization framework, in this case, the EMA. Several opportunities for including production-related environmental considerations in the European approval process exist (Ågerstrand *et al.*, 2015; Nijsingh *et al.*, 2019), leaving room for national agencies to act within that remit. While the feasibility and consequences of an environmental incentivizing mechanism may depend on whether it is implemented at a national or a multinational level, the impact of actions taken at these levels is potentially high compared to actions taken at local or regional levels.

In the authorization context, using environmental criteria as eligibility conditions will make market approval contingent on fulfillment of the criteria, effectively closing legal access to a national (or, in the EMA case, multinational) market for noncompliant suppliers. A gradual weight approach, in contrast, will entail balancing environmental criteria against other considerations, in this context primarily clinical effect, evidence-quality and patient safety. How such trade-offs are made will in turn determine the exact implications of this model.

### Public Procurement

Drug procurement by healthcare providers offers opportunities to include environmental aspects among quality requirements on procured products. Since 2010, Swedish county councils have a joint agreement on sustainability criteria intended to be included in contracts with suppliers, including a requirement of compliance with environmental law in production countries. Compliance with the criteria can be subject to auditing and noncompliant suppliers risk sanctions ranging from a fine to annulment of the contract (Hållbar upphandling, 2019). However, there is no central audit of how rigorously county councils implement the agreement, and each county council is responsible for ensuring that the conditions are actually included in contracts and fulfilled by suppliers. Moreover, demanding compliance with producer country environmental legislation may have little impact if this legislation itself is lax. Besides this agreement, in October 2019 UM published a set of recommended environmental criteria for procurement of pharmaceuticals, including requirements of disclosure of subcontractors’ geographical location and of risk management related to API discharges (UM, 2019). However, county councils remain free to decide which (if any) of these criteria to include in contracts and they have, to our knowledge, not yet been used. In the Swedish case, procurement decisions have limited impact as they are made at a regional level, although recent initiatives attempt to create more harmonization and centralized control (Nijsingh *et al.*, 2019). In general, more centralized (i.e. national or multinational) procurement systems have stronger potential for forceful impact.

In this context, designing environmental criteria as eligibility conditions will create thresholds that products available on the market must pass to be considered for procurement by a public actor, regardless of their features in terms of cost, effect, patient need, evidence, etc. If, instead, they are designed as gradual weights, their fulfillment will be one of several features a public procurer considers when setting healthcare priorities and allocating resources for drug purchase based on that. Besides effect, safety and evidence basis, these features typically include the cost of drugs under consideration, the needs of different patient groups for which drugs are being considered for purchase, the availability of alternative treatments to meet these needs and legal requirements of prioritizing only on the basis of need and of equal treatment of equivalent needs (Prop. 1996/97:60).

### Public Subsidy

Systems for subsidizing pharmaceuticals may substantially influence procurement decisions, regardless of how the latter are organized, but also the consumption of prescription drugs sold to consumers via pharmacies. The Swedish agency responsible for subsidy decisions, TLV, does not presently consider environmental criteria in these decisions, but focuses on cost-effectiveness, reflecting its overall aim of making the most of public resources available for drugs. In addition, TLV observes the same legally mandatory ethical principles for fair priority setting that guide procurement decisions of public healthcare institutions. However, TLV is tasked with not only calculating the individual cost–benefit ratio (in terms of cost per QALY), but also assessing cost-effectiveness from a societal standpoint, thereby determining the ‘societal consumer preference’ for new drugs.^4^ In that context, some weight is already given to the societal value of increasing individuals’ working capacity (which may offset the initial cost by reducing future cost) and of solidarity with so-called orphan disease patient groups (TLV, 2020b). As ABR threatens the future effectiveness of healthcare, including environmental criteria when assessing subsidy for antibiotics could be considered another way of promoting healthcare system sustainability by protecting the public interest in effective antibiotics and the ethical value of equal treatment of present and future patients.

Applying environmental criteria as eligibility conditions in this context will mean entirely barring drugs that do not fulfill the criteria from public subsidy, whatever other qualities in terms of cost, effect, patient need, evidence, etc. they possess. Such drugs will still be available on the market and for public procurement, but at a much-elevated price. Drugs that pass this threshold will be considered for subsidy, but without any further attention to environmental concerns. If environmental criteria are instead used as gradual weights, the degree to which they are fulfilled will be considered alongside the factors of cost-effectiveness and ethics that influence whether a drug is subsidized. This means that environmental concerns may be outweighed by large patient need, good effect or low price, but also that grave environmental concerns may preclude subsidy despite, e.g., good effect and low cost.

### Generic Substitution

It has been observed that any incentive for the pharmaceutical industry to address its environmental problems has to apply equally to all industry actors (Nijsingh *et al.*, 2019). Thus, when a publicly funded healthcare system applies mechanisms for generic substitution of subsidized drugs to increase cost-effectiveness, actions targeting the licensing or subsidy institutions must also include their role in the generic substitution system. Otherwise, the created incentives will only affect research-based manufacturers (‘brand’ companies), and only as long as their patents hold. In effect, generic antibiotic manufacturers who decrease costs by ignoring environmental concerns would gain a competitive advantage over both brand companies and generic companies that (voluntarily) fulfill environmental criteria (Bengtsson-Palme *et al.*, 2018). Conversely, inserting environmental criteria into the generic substitution system *only* would be rather pointless, as that would give a competitive advantage to brand manufacturers who ignore environmental concerns. Rather, any action toward this system must also address its counterpart in the licensing or subsidy stem (or both).

The Swedish generic substitution system presents two distinct routes for influencing the generic pharmaceutical industry to address industrial antibiotics pollution: (i) assessment of interchangeability and (ii) selection, among interchangeable drugs, of generic alternatives that pharmacies must offer to customers. Regarding the first task, which is handled by LV, including environmental considerations in interchangeability criteria would constitute a significant modification, as current criteria are exclusively concerned with ensuring identical effects on individual patients (LV, 2019). Preferably, an implemented revision would assign equal importance to environmental considerations in the licensing process for branded drugs and in the interchangeability assessment process for their respective generic counterparts.

Regarding the second task, handled by TLV, including environmental considerations when selecting recommended generic alternatives would also significantly depart from current practices, which almost exclusively focus on price. This focus is based on the rationale that because a subsidy assessment has already been made of the branded drug, and the generic alternative has been declared interchangeable to that one, no new subsidy assessment is needed for the latter. However, as the branded and generic alternatives may differ significantly with regard to environmental criteria, this rationale would crumble once such criteria are taken into consideration. We argued above that the TLV charter may well mandate environmental considerations to enter the subsidy assessment process, and if they do, the same should hold for the selection of recommended generic alternatives.

Again, for both routes, environmental criteria can be designed as eligibility conditions or as gradual weights. For the LV pathway, the choice of role should reflect the role of environmental criteria in the licensing system. If a generic drug fails to meet an environmental eligibility condition, the drug will be barred from the interchangeability assessment process, whatever its other features. With a gradual weight design, the environmental profile of a generic drug will instead be balanced against other considerations relevant to licensing—i.e. efficacy, safety, quality and evidence supporting these. For the TLV pathway, the choice of role should instead reflect its counterpart in the subsidy system. An eligibility condition design will imply that interchangeable generic drugs that fail to meet environmental criteria are barred from being recommended to pharmacies as alternatives to branded drugs, while a gradual weight design will mean that the selection of recommended generic alternatives will balance environmental considerations against other features—in this case, presumably mostly price.

## Tensions and Trade-Offs

The preceding section described several opportunities for actors within public healthcare institutions to address industrial antibiotic pollution. This section continues the analysis by demonstrating that pursuing any such opportunity will create tensions between different objectives of such institutions. Specifically, tensions are liable to arise between two or more of the following aims:

1. keeping effective antibiotics on regional, national or multinational markets,
2. ensuring that patients receive the antibiotics that are in their best clinical interest,
3. ensuring patients’ equal access to effective antibiotics based on health need,
4. limiting societal spending on antibiotics (and drugs in general), and
5. reducing pollution from antibiotic manufacturing.

Each of these aims carries significant normative weight for public healthcare institutional actors.^5^ We argued above that these actors ought to address antibiotics pollution (aim 5) because of their capacity to do so, their contribution to the problem, and their overarching concern for the health of a designated population. On the other hand, the last consideration also gives the actors reason to seek to promote patients’ access to effective antibiotics (aims 1 and 2). The ethical rationale of healthcare, moreover, rests on patients’ interest in receiving needed drugs, which adds a requirement of equal access based on equal need (aim 3). Furthermore, the concern with protecting and promoting health generates reasons for keeping the price of antibiotics down (aim 4), since society’s budget for pharmaceutical spending is limited and increased spending will require foregoing spending on treatments for other patients. None of these aims can reasonably be ignored, but, as we shall now see, 1–4 cannot be completely fulfilled simultaneously with 5.

Tension between 1 and 5 arises due to the nature of business: any action that makes it more costly and/or difficult to enter a market, sell products for profit on that market, etc., creates an incentive for a company to avoid that market and instead spend its resources where business prospects are more attractive.^6^ This tension is especially pronounced with regard to actions taken by authorization institutions, as these directly influence the basic conditions for market entry. However, as procurers’ and subsidy institutions’ actions will require companies to make efforts or incur costs, thus indirectly affecting their prospects for meeting their return expectations, these actions too create tensions between 1 and 5.

Tension between 2 and 5 arises partly through the first tension mentioned above: if effective antibiotics do not enter a market, patients that depend on what is offered in that market are barred from accessing them. Similarly, if an antibiotic is denied subsidy, it will be more expensive and thus less affordable to patients and less likely to be procured by a public healthcare system. And if a drug is not procured, either because of its price or directly because of environmental concerns, most patients will not be able to access it. Insofar as the unaffordable or unavailable antibiotic is, from a clinical perspective, the best treatment for at least some patients, aim 2 will have been set aside to some extent.

Tension between 3 and 5 is in turn related to the second tension just described. Some patients may still be able to afford non-subsidized antibiotics or to access antibiotics outside of the public healthcare system, e.g. through private providers or international contacts. However, access to these drugs will not be equal or based on need. In addition, as procurement decisions that directly or (due to lack of subsidy) indirectly reflect environmental concerns may vary between public healthcare actors—in Sweden, different county councils—a further inequality of access may result.

Tension between 4 and 5 becomes salient as we ponder how the other tensions may be addressed through increased public spending. Companies may be more willing to enter markets despite demanding environmental conditions if the public healthcare system compensates them financially, e.g. through pricing mechanisms, an adjusted procurement preference, or in some other way. Likewise, the procurement preference may be adjusted to compensate for the costs incurred by meeting environmental criteria in order to secure patients’ equal access to effective antibiotics. Ultimately, however, the outcome will have to be increased drug procurement costs in the antibiotics area, thus conflicting with more general aims of healthcare systems to curb expenses. A particular effect of this kind follows from actions targeting the generic substitution system, as the point with this system is to exploit the availability of cheap generic alternatives to decrease pharmaceutical spending. Actions restricting the availability of such alternatives will thus undermine the economic impact of the system. At the same time, we have seen that any action targeting the authorization or subsidy institutions must also include the generic substitution system in order to effectively incentivize pollution management.

These tensions are unavoidable but may be handled differently and be more or less severe, depending on how environmental criteria are formulated and used by different actors. Here, the distinction between eligibility conditions and gradual weights becomes particularly important. In general, eligibility conditions create a binary situation as regards the consequences of meeting or failing to meet the criteria: an antibiotic is either completely excluded (from license, subsidy or procurement), or unconditionally available (for the same). This approach would ensure that drugs that remain available within a given system actually fulfill basic environmental criteria. Also, its simplicity might make it relatively easy to administer and difficult to manipulate. However, there are two notable disadvantages. First, patients’ access to excluded drugs would be barred or significantly impeded. Second, eligibility conditions are insensitive to the *degree to which* environmental criteria are fulfilled. Improving a drug’s environmental record makes no difference to its status if it remains below the threshold, and once a drug reaches the threshold further improvement makes no difference either. Thus, this approach would have little impact on companies that already reach the threshold or are only prepared to make improvements falling short of it.

The requirement that suppliers comply with environmental law in production countries currently applied in the Swedish procurement context illustrates the eligibility condition approach. This requirement would, if strictly monitored and enforced, effectively bar noncompliant suppliers from selling their products to county councils. Also, compliant suppliers are not positively rewarded or further ranked based on environmental performance. In contrast, the Norwegian procurement organization, *Sykehusinnkjøp*, is currently implementing a model of gradual weights which selects suppliers of pharmaceuticals based on a weighing of cost-effectiveness (50 per cent), environmental performance (30 per cent) and supply reliability (20 per cent) (LMI, 2018).

Since the Norwegian model affords increasing priority to actors proportional to how well they meet environmental criteria without barring any actor, it illustrates how a gradual weights model is less prone to sacrificing access to antibiotics. However, the tensions between aims 1 and 5 do not thereby disappear. Such a model allows suppliers to compete along several different dimensions, in the Norwegian case, cost-effectiveness, supply reliability and environmental performance. Clearly, it cannot be expected that suppliers scoring well on one dimension necessarily score well on the other two. Thus, in this case, environmental performance will have to be paid for in terms of higher price, reduced effectiveness or lower supply reliability, or, conversely, environmental performance will have to be sacrificed in favor of the other dimensions. Here three objectives—controlling pollution, limiting drug spending and ensuring that patients receive optimal treatment—pull in different directions.

The role of eligibility conditions may play out significantly differently in relation to different institutional functions. In authorization, eligibility conditions would require companies to choose between striving to meet the criteria or abstain entirely from entering the market in question. If this market is relatively small, it may not make good business sense to spend resources to enter it—especially if larger markets offer laxer conditions. In a larger national or (if authorization is harmonized across countries) multinational market, business reasons may instead motivate companies to produce products complying with demanding criteria. If eligibility conditions are instead applied in public procurement (directly or via the subsidy system), they would have a similar effect on companies’ willingness to sell their products to health system actors. The magnitude of the effect would presumably be smaller here since access to one or a few buyers rather than an entire national or multinational market is at stake, and would also depend on the business opportunities available to companies outside of publicly funded healthcare. In both systems, conflicts would arise between aim 5 and aims 1–3, and these would be quite drastic due to the binary nature of eligibility conditions. Moreover, in both cases, market competition would drop, presumably driving prices up and creating a tension with aim 4. Environmental criteria could of course be made relatively undemanding to avoid sacrificing aims 1–4, but this would hamper the pursuit of aim 5 instead.

A gradual weight approach may soften these tensions by allowing companies to escape environmental requirements by excelling in terms of, e.g. quality, price and supply reliability. The obvious drawback is that environmental impact is not guaranteed since companies may seek competitive advantage in other ways than by reducing pollution. Moreover, a weight approach might clash with aim 2, since including environmental concerns as a further factor to consider, besides quality, price, etc., would allow two drugs with identical cost-effectiveness targeting equally needy patient groups to receive different verdicts due to different environmental performance. But this tension, as observed, arises for an eligibility condition approach too, the difference being that a weight approach leaves room for institutional actors to balance the different factors and adjust their respective weights to find justifiable equilibria. The room for companies to adjust is thus mirrored by a similar room for society to finetune policy with regard to all the aims.

Since none of the aims 1–5 can be reasonably ignored and since they conflict with each other, trade-offs between them are unavoidable. These trade-offs are necessary for policy decisions concerning (i) what institutional actor(s) shall apply environmental criteria, (ii) whether criteria shall be designed as eligibility conditions or as gradual weights and (iii) what substantial requirements these criteria shall make. Importantly, however, such trade-offs are not only necessitated by potential policy changes aimed at addressing industrial antibiotics pollution. The current status quo—where very little weight is attached to this problem—also represents a set of trade-offs, albeit ones where the aims 1–4 take precedence over 5. The question, then, is not whether to make trade-offs but how to make them in *defensible* ways. In the next section, we probe a number of underlying ethical issues that must be tackled to approach such justification.

## Underlying Conflicts: Ethics and Pragmatics

Reaching defensible trade-offs between aims 1–5 is a complex task. From a principled perspective, the trade-offs should be based on compelling moral principles, which give due weight to each of the aims and the values underpinning them, and justify particular ways of balancing these. Otherwise, the resulting policy lacks sound ethical basis. But in addition, recommendable trade-offs must be pragmatically justified, so that they are capable of working in practice. Among other things, they must enjoy sufficient long-term social and political legitimacy for individuals and institutions to be motivated to make the requisite structural and behavioral changes and tolerate the burdens these changes involve. Principled and pragmatic considerations are not always in harmony, however, and the ABR case illustrates this clearly (Nijsingh *et al.*, 2019; Munthe and Nijsingh 2019; Munthe *et al.*, 2019). From a principled perspective, implementing rather drastic measures to curb ABR may seem justified, given the scale and urgency of the problem. But from a pragmatic perspective, such measures risk being perceived as excessively onerous, provoking social and political resistance obstructing their implementation (as well as perhaps fostering black markets in antibiotics). Reaching defensible trade-offs capable of motivating institutional action therefore also requires balancing principled and pragmatic considerations.

We will now explicate several fundamental ethical conflicts that underlie the policy tensions described in the previous section.^7^ Our argument is that, on closer analysis, no particularly controversial position on these underlying conflicts seems needed to endorse prioritizing the policy goal of addressing pollution, but that implementing this principled stance would create pragmatic challenges which defensible action must accommodate.

### Here Versus There

As noted above, each of the five objectives has significant normative weight for the relevant actors because of their concern with promoting and protecting health and well-being. However, 5 is special in that the immediate health gains of achieving it would occur in API producer countries (e.g. India), whereas populations in high-income countries where the final product is consumed (e.g. Sweden) would benefit more indirectly. By contrast, 1–4 primarily serve the interest of the population in a given consumer country. Thus, the question of trade-offs is in part a question of weighing local health gains against health gains occurring in another part of the world.

The underlying issue here concerns the moral relevance of special relationships, particularly relationships of nationality or citizenship—an issue dividing consequentialists from nonconsequentialists in ethics, and cosmopolitans from nationalists in political philosophy (Scheffler, 1997; Miller, 2005; Pogge, 2008). Despite fundamental disagreement on this issue, however, there is room for convergence on the view that health gains matter morally regardless of where they occur. This is because most defensible normative moral and political theories agree that morality requires adopting an impartial point of view (Wolf, 1992). While the exact interpretation and practical implications of this idea are debated, there is broad agreement that moral agents owe at least a basic level of concern to *all* persons, irrespective of any relationship or past interaction with them. From this perspective, the fact that the immediate harms of antibiotic pollution and the immediate health benefits of reducing it accrue to people in another country is no reason to ignore these harms and benefits. Rather, assuming that their respective magnitude is significant, it appears that actors in consumer countries should give them considerable weight.

Of course, the difficulty here is that many moral theories (as well as common sense morality) recognize both natural duties, which are owed to everyone impartially, *and* special obligations arising from relationships or voluntary commitments (Scheffler, 1997). Thus, it might be proposed that a state’s responsibility concerning the health of its population (reflected in aims 1–4) is best understood as a set of special obligations that hold between fellow citizens or compatriots (Miller, 2005). Furthermore, it might be proposed that these obligations trump whatever duty that citizens or state institutions acting on their behalf may have regarding the health of non-citizens—just like somebody’s special obligation to provide their child with a decent education might be thought to trump a natural duty to assist needy strangers if this person cannot do both. The upshot is that institutional actors in consumer countries should prioritize local health interests over health interests in producer countries.

In response, note that philosophical defenders of national partiality do not normally hold that special obligations to compatriots *always* trump global natural duties (Dagger, 1985; Miller, 2005). In particular, duties to refrain from harming innocent people anywhere or infringing their basic rights cannot be outweighed by local obligations, even obligations of justice (Miller, 2005). Thus, if institutional actors in consumer countries currently contribute to harmful antibiotic pollution in producer countries (as argued above), then they have weighty reasons to address this problem even at the cost of somewhat forgoing advancing local health interests. And even if this argument is rejected because the assumed causal connection is hard to establish, defenders of national partiality would support prioritizing addressing pollution insofar as they recognize a global duty to transact fairly that normally overrides special obligations to compatriots (Miller, 2005). For the current international system of antibiotics production may plausibly be considered distributively unfair as the resulting health and economic benefits accrue to rich and poor countries alike whereas the burdens of pollution are disproportionately allocated to the latter.

Thus, defenders of national partiality have reason to grant that institutional actors in consumer countries should attach significant weight to reducing antibiotic pollution in producer countries. This would be in agreement with cosmopolitan-oriented theories, according to which the fact that the benefits of addressing pollution would accrue to foreigners is no reason to disregard or discount those benefits in the first place. Hence, the same practical conclusion can be reached despite underlying philosophical disagreement concerning global justice and national partiality.

### The Present Versus the Future

The problem under consideration has a temporal as well as a spatial dimension. If an institutional actor in a consumer country pursues the goal of reducing antibiotics pollution in producer countries at some cost in terms of advancing aims 1–4, some people in that country will suffer potential burdens. They may, for instance, risk not receiving optimal antibiotic treatment, or enjoy a smaller portion of healthcare resources than they otherwise would. By contrast, the potential benefits of this policy (to individuals in the same population) will mostly materialize at a later point in time. Admittedly, preserved effectiveness of antibiotics will likely generate some fairly immediate benefits. However, since most antibiotics are not at risk of immediately becoming totally ineffective and since pollution in a producer country presumably affects resistance in a consumer country only with some delay, promoting aim 5 would primarily serve the long-term interests of the relevant community. Thus, the question of a trade-off is partly a question of weighing short-term burdens against long-term benefits (again to individuals in the same population).

Considerations of impartiality of the sort discussed above seem like a plausible starting point for approaching this issue (Millar, 2011). Temporal distance in itself seems no more ethically relevant than spatial distance. Other things being equal, the fact that some harm or benefit will accrue to one person later rather than to another person sooner does not seem to make it less (or more) significant. So, on the face of it, the relevant actors appear to have reason to let the long-term benefits of reducing antibiotics pollution outweigh the short-term burdens of somewhat setting aside their other goals, at least insofar as the benefits are of greater magnitude.

The difficulty is that other things are often *not* equal when we compare temporally proximate and distant harms and benefits. In particular, vexing problems arise if the temporal scope is extended to include future generations. There is deep philosophical disagreement concerning whether not yet existing people can have moral claims on existing ones and, if so, what the nature and stringency of those claims are (Parfit, 1984; Gosseries, 2005). These disputes over intergenerational ethics and justice clearly appear to complicate any attempt to justify prioritizing securing the potential long-term benefits of reducing pollution over avoiding short-term burdens.

However, such priority can be defended without taking sides in the underlying philosophical disputes. The existence of effective antibiotics is to a large extent a *precondition* for the actors’ successful long-term pursuit of the four other goals. It clearly makes little sense to keep antibiotics on the market (aim 1) or ensure equal access to them (aim 3) if they no longer work. Similarly, ensuring that patients receive optimal antibiotic treatment (aim 2) becomes more difficult the more the effectiveness of these drugs erode due to pollution. And society’s health-related costs will surely soar (compromising aim 4) if infections cannot be treated, or only treated less effectively and with more severe side-effects, and if advanced procedures can no longer be safely performed. Thus, insofar as these other objectives matter to the actors they have strong indirect reasons to counteract resistance development, e.g. by addressing antibiotics pollution.

Similarly, it might be argued that the actors should care about curbing ABR out of concern for their own functioning over time. These actors are essentially concerned with protecting and promoting the health of a designated population. They should therefore also be concerned with their own ability to continue protecting and promoting health, hence also with securing the preconditions of that ability, including the existence of effective antibiotics, without which the pursuit of their constitutive goal is significantly hampered. Sustainability considerations of these two kinds support attaching some priority to the long-term aim of reducing antibiotic pollution without assuming any particular theory of justice and ethics across generations.^8^

### Individuals Versus Collectives

Yet another dimension of the problem concerns collective and individual interests. Everyone stands to benefit from the ability to treat infections: not only the infected individual but also the wider community (Selgelid, 2007). Any intervention that contributes to slowing resistance development, including interventions that effectively reduce production-related pollution (aim 5), helps protecting this collective interest. By contrast, some of the competing objectives primarily serve the interest of an individual receiving treatment. Specifically, aim 2 sometimes effectively constrains the relevant actors’ pursuit of other ends. For instance, the Swedish generic substitution system only allows reduced pharmaceutical spending to be pursued among clinically interchangeable drugs. Thus, the collective interest in maximizing resource use can never override the individual’s interest in receiving optimal treatment. The same interchangeability criterion would (if left unchanged) place an analogous constraint on attempts to realize the collective goal of reduced antibiotic pollution in this system, considerably limiting the effectiveness of any such attempt. As this example illustrates, ABR raises questions about the legitimacy of established individualist moral constraints in situations where critical public interests are at stake (Selgelid, 2007; Millar, 2012; Littmann and Viens, 2015; Rid *et al*., 2019).

One approach here is to tackle the underlying conflict head on by relaxing the individualist constraints when the collective interests are sufficiently large and urgent. In the ABR case, one could defend such an approach by drawing an analogy to other contexts where individualist constraints are legitimately loosened for the common good. For instance, physicians’ conduct is traditionally understood to be constrained by the obligation to act in the individual patient’s best interest. However, in many situations, including physician training, clinical research and infectious disease management, this obligation is justifiably somewhat set aside for the sake of important collective interests, e.g. in skilled healthcare staff, medical knowledge and infection control (Wendler, 2010). It might be argued that the individualist interchangeability criterion in the generic substitution system may also justifiably be relaxed to promote the no less important collective interest in reducing antibiotic pollution.^9^

A more moderate approach is to sidestep the underlying conflict by targeting systems where individualist considerations do not constrain the pursuit of collective interests. For instance, in the Swedish public subsidy system, the patient’s interests in receiving optimal treatment do not impose any strict limit on society’s efforts to control spending on drugs. Clearly, society will not pay just *any* amount for a new drug that is slightly better than the alternatives. Rather, the two interests are weighed against each other, and the cost-effectiveness threshold (specified as a certain sum per QALY gained) represents the point where an appropriate balance is taken to be struck. Effectively pursuing the goal of reduced antibiotic pollution within such a system would not require relaxing individualist moral constraints for the common good. Instead, the weighing of individual and collective interests would be complemented by another collective interest, namely, in curbing resistance development. Of course, attaching weight to this consideration would entail correspondingly reducing the weight given to one or both of the other considerations. But this would not drastically depart from the prevailing normative logic of the system.

### Pragmatic Challenges

Without assuming any particularly controversial normative position, we have argued that high-income state actors have strong moral reasons to address production-related antibiotic pollution and to attach some priority to this objective over advancing their other goals. However, while the principled case in favor of this stance seems compelling, translating it into concrete action raises new challenges. Since ABR is an extraordinarily complex problem involving numerous actors and institutions with conflicting aims and interests on different levels (Littmann *et al.*, 2020), careful attention must be paid not only to the formal justification of policies, but also to their feasibility and social sustainability in light of different parties’ reactions to the consequences of rolling them out (Nijsingh *et al.*, 2019; Munthe and Nijsingh, 2019; Munthe *et al.*, 2019). Such reactions may lead to severe nonadherence, reduced legitimacy, and, in the worst case, long-term blocking of entire ranges of political action (Munthe and Nijsingh, 2019).

In the present context, we will highlight two particular dimensions of pragmatic concerns. First, the tensions between the aims 1–5 themselves may well actualize drastic practical conflicts between the content of recommended policy and key actors involved in implementing it. For instance, aims 2 and 3 incorporate central tenets of clinical ethical standards, potentially limiting the willingness of clinical health professionals to accept that these standards are lowered to accommodate aim 5. Another example is strong political commitments to minimizing public expenditure (fuelled by voter pressure for tax reduction), which may make policymakers unwilling to prioritize aim 5 over aim 4 in the way necessary to create effective incentives for industry. Both these examples also illustrate how trade-offs between the aims need to consider pragmatic factors when the exact policy is designed.

The other pragmatic dimension links to the fact that any positioning on the underlying ethical conflicts is bound to be controversial. Besides complicating principled justification of trade-offs between the aims 1 and 5, this means that any trade-off may encounter significant resistance grounded in underlying ethical disagreement. In particular, through all of the conflicts between the aims run a general division between ideals of universal inclusion (regardless of place, time or social situatedness) and ideals of partiality toward particular interest or stakeholder types (our population, my patient, individuals over groups), where fundamental and strong disagreements are known to prevail. Policy compromises seeking to balance the aims based on some principled perspective will therefore need to consider how to accommodate competing perspectives to ensure feasible implementation and political sustainability. Our observation above that different principled perspectives may support similar balancing indicates some room for common ground, but political recognition of this common ground may itself be hard to achieve.

## Conclusion

Reducing industrial antibiotics pollution is increasingly recognized as an important goal for responsible ABR policy. We have explored the role of institutional actors within publicly funded healthcare systems in high-income countries in achieving this goal. We have argued that these actors have strong ethical reasons and realistic opportunities to take action to incentivize industry to reduce antibiotic pollution. However, any institutional action in this area would create tensions with other significant policy goals, necessitating trade-offs. Analyzing fundamental ethical conflicts underlying these tensions, we have presented a principled case for attaching some priority to addressing pollution over competing goals, while also emphasizing pragmatic considerations complicating any simple implementation of this principled stance. With pragmatics in mind, sacrificing the full realization of principled commitments in favor of more feasible and sustainable approximations may be defensible based on these commitments themselves.

A key result of our analysis concerns the different implications of designing incentives as eligibility conditions and as gradual weights. The former approach attaches central importance to reducing antibiotic pollution, but would require drastic compromises with other policy goals, especially that of ensuring access to antibiotics. The latter approach permits more nuanced balancing of competing goals, but without guaranteed impact on pollution. Different designs may suit different context and hybrid models are conceivable. However, a gradual weight design would often seem preferable for pragmatic and principled reasons: it would soften tensions between the aims and interests of different stakeholders, facilitating the implementation of sustainable policy, and recognize the distinct values underpinning different policy goals.

A limitation of our analysis is that the specific examples of policy reform considered focus on Swedish systems and actors. However, the *institutional functions* (authorization, subsidy, procurement and generic substitution) illustrated by these examples are present, in some form, in other high-income countries with publicly funded healthcare systems too. Differences in policy priorities and health system organization may nevertheless mean that different concrete policies are conceivable in different contexts. Future research should therefore consider opportunities and ethical challenges for institutional action on industrial antibiotic pollution in other national as well as international settings. Our broad overview of opportunities and challenges in this area may hopefully pave the way for such work.

## Funding

This work was supported by the Swedish Research Council (grant number 2018-05771); Vinnova (Sweden’s Innovation Agency) (grant number 2018-00433); and the UGOT Challenges Initiative of the University of Gothenburg.

## Conflict of Interest

None declared.

## Footnotes

1 Emissions via urine and feces of humans and animals who use antibiotics are widespread, too, but it is unclear whether the concentrations involved are high enough to select for resistant bacteria (Karkman *et al.*, 2019).

2 Since antibiotic use in healthcare and meat production creates an externality in the form of increased resistance, fees or taxes that require consumers or producers to internalize this cost may be defended analogously (Anomaly, 2013, 2015; Giubilini *et al.*, 2017; Giubilini, 2019).

3 For further details and additional actors, see Nijsingh *et al.* (2019).

4 This holds for similar agencies in other countries too, e.g. NICE in the UK.

5 The actors we discuss may have other normatively significant aims in addition to the ones listed here, aims that may also conflict with the goal of reducing pollution. Moreover, the listed aims may have significance other public actors too. For instance, research funders plausibly have reason to pursue 2 and 4 by supporting the development of new therapies and diagnostics.

6 This is not idle speculation. Medically important antibiotics are commonly unavailable on national markets in developed countries, the main reason for withdrawal being lack of profitability (Pulcini *et al.*, 2012).

7 While the underlying conflicts are familiar from other policy responses to ABR (Selgelid, 2007; Millar, 2011, 2012; Littmann and Veins, 2015) and from public health policy more broadly, they are, as we shall see, manifested in specific ways with regard to action on industrial antibiotic pollution. Notably, the list of conflicts considered is not exhaustive. Another conflict, outside of our focus on healthcare systems, concerns human and nonhuman interests. Since antibiotic pollution potentially harms wild animals and ecosystems, ethical views that attribute moral status to these entities (see Brennan and Lo, 2016) may support prioritizing reducing the pollution over other aims. More drastically, though, since some of these views give little weight to the human interests targeted by these aims (including aim 5) and since ABR results from an evolutionary process whereby an ecosystem maintains its equilibrium, they may not consider the pollution a problem at all, nor regard the human-centered tensions between aims 1 and 5 as ethically significant.

8 Of course, this argument assumes that future generations matter morally *in some sense*. But this is usually not denied by participants in these debates (Gosseries, 2005).

9 Similarly, one could defend programs for ‘rational’ clinical antibiotic use that impose some risk on individual patients to secure an important public health benefit by appealing to an analogous risk-benefit trade-off in clinical research (Rid *et al.*, 2019).
